# Book Notices

**Published:** 1860-11

**Authors:** 


### Book Notices.

PUBLIC SCHOOL SINGING-BOOK, by Prof. John Bower, teacher of music in the Public Schools of Philadelphia, is another step towards making us a musical people. Music and song purify the heart and the blood also; their culture never fail to elevate, under propitious influences.

REASON AND THE BIBLE. By Miles P. Squier, D.D., Prof. of Intellectual and Moral Philosophy, Beloit College, Wisconsin. Scribner, Publisher, 124 Grand street, New-York, is founded in the thought that "Reason leads to Faith." 340 pp. 12mo, large type, $1. The reading of this book will sweetly confirm the humble believer, will establish the wavering, and we trust, will convince many a "philosophical" doubter that there is no firmer "foundation" than that laid in the Rock of Calvary. To every doubting, halting man or woman, we heartily say, get it and read it.

THE ATTORNEY. Irving. Dewitt, Publisher, is one of the most intensely interesting narrations we ever found in the pages of the *Knickerbocker*, where it first appeared. The scenes are laid in New-York City. The book is worth a million of the trashy, flash productions of the times, and we trust the enterprising publisher will find his reward in thus wisely drawing from the stronger and purer fountains of the past.

HARRY HARSON, by the same author, and published by the same house, will, we think, be inevitably read by every man who has turned the all-absorbing pages of THE ATTORNEY.

THE MOVEMENT CURE.—The most sensible "movement" that has come to our knowledge for a long time. Dr. Taylor has written an interesting and useful book, and extends the application of the views of Dr. Halsey, his predecessor. As we are anxious to benefit our readers whenever it is in our power, we will condense it for those who may not be inclined to pay a dollar for so sensible a book. Our understanding of it is this: If you want to get well, go to work.

BERIAH GREEN'S SERMONS and DISCOURSES, with Brief Biographical Hints, 12mo, 556 pp. S. W. Green, 18 Jacob street, New-York, Publisher. With a life-like engraving. The Rev. Beriah Green is one of the men of our time; earnest, practical, and persistent, he has, with the advantage of a clear head, a strong mind, and a good heart, done more for true progress than many who have made more noise and gained more of the world's applause.

DAUGHTERS.—Our Kentucky readers—and we have a good many—are notified that the Oxford Female College, an hour's ride by rail from Cincinnati, O., under the presidency of the Rev. Mr. Morris, is in most successful operation. President Morris is himself a Kentuckian in the highest and widest sense; frank, courteous, and hospitable. He possesses one of the finest minds in the West. High culture is united with untiring and indomitable energy. To be under his roof, and under the influences of his lovely and accomplished wife, is a privilege which is to be enjoyed by few, and parents who must send their daughters from home to be educated, will act wisely by securing it at once.

All our publications may be had of Mr. Mowry, at the Post-Office entrance, Philadelphia, and also of John McFarlan, 33 South Sixth street, in same city.

Please read the leader of the present number, on Periodical Literature; meanwhile we claim for the JOURNAL OF HEALTH, that it is one of the very few Monthlies, not professedly religious, which has never sought to make capital for itself by ridiculing religion, its ministers or its friends, but has always and decidedly, advocated the Christianity of the Bible, and in these regards merits the patronage it receives. We ask an equally wide one for our other monthly, THE FIRESIDE.

